# Influential Nodes Identification in Complex Networks via Information Entropy

**DOI:** 10.3390/e22020242

**Published:** 2020-02-21

**Authors:** Chungu Guo, Liangwei Yang, Xiao Chen, Duanbing Chen, Hui Gao, Jing Ma

**Affiliations:** 1 School of Computer Science and Engineering, University of Electricity Science and Technology of China, Chengdu 611731, China; thespringguo@gmail.com (C.G.); y805797986@gmail.com (L.Y.); ahuigao@gmail.com (H.G.); 2 Information Assurance Office of Army Staff, Beijing 100043, China; 18215561894@163.com; 3 The Center for Digital Culture and Media, University of Electricity Science and Technology of China, Chengdu 611731, China; 4 Institute of Fundamental and Frontier Sciences, University of Electricity Science and Technology of China, Chengdu 611731, China; 5 Union Big Data Tech. Inc., Chengdu 610041, China; 6 Business School, Sichuan University, Chengdu 610064, China; jingma@stu.scu.edu.cn

**Keywords:** complex networks, influential nodes, information entropy, SIR model

## Abstract

Identifying a set of influential nodes is an important topic in complex networks which plays a crucial role in many applications, such as market advertising, rumor controlling, and predicting valuable scientific publications. In regard to this, researchers have developed algorithms from simple degree methods to all kinds of sophisticated approaches. However, a more robust and practical algorithm is required for the task. In this paper, we propose the EnRenew algorithm aimed to identify a set of influential nodes via information entropy. Firstly, the information entropy of each node is calculated as initial spreading ability. Then, select the node with the largest information entropy and renovate its *l*-length reachable nodes’ spreading ability by an attenuation factor, repeat this process until specific number of influential nodes are selected. Compared with the best state-of-the-art benchmark methods, the performance of proposed algorithm improved by 21.1%, 7.0%, 30.0%, 5.0%, 2.5%, and 9.0% in final affected scale on CEnew, Email, Hamster, Router, Condmat, and Amazon network, respectively, under the Susceptible-Infected-Recovered (SIR) simulation model. The proposed algorithm measures the importance of nodes based on information entropy and selects a group of important nodes through dynamic update strategy. The impressive results on the SIR simulation model shed light on new method of node mining in complex networks for information spreading and epidemic prevention.

## 1. Introduction

Complex networks are common in real life and can be used to represent complex systems in many fields. For example, collaboration networks [1] are used to cover the scientific collaborations between authors, email networks [2] denote the email communications between users, protein-DNA networks [3] help people gain a deep insight on biochemical reaction, railway networks [4] reveal the structure of railway via complex network methods, social networks show interactions between people [5,6], and international trade network [7] reflects the products trade between countries. A deep understanding and controlling of different complex networks is of great significance in information spreading and network connectivity. On one hand, by using the influential nodes, we can make successful advertisements for products [8], discover drug target candidates, assist information diffusion [9], and even detect essential proteins [10]. On the other hand, by removing some critical nodes, it can greatly reduce the connectivity of the network to restrain the outbreak of epidemics [11] or spreading of rumors [12].

The ongoing COVID-19 epidemics is catching wide attention around the world. Every country is making enormous effort to control the virus spreading. By analyzing social networks, it would be easier for us to control epidemics spreading. In the last decades, propagation dynamics has always been an important research direction. Many mechanisms, such as epidemic spreading [13,14,15,16], rumor propagation [17,18], social sudden events spreading [19], and e-commercial advertisements, are all closely related to complex network dynamics. Early in 1760, Daniel Bernoulli studied smallpox vaccine by using ordinary differential equations, and gave the Bernoulli equations [20], which is one of the earliest propagation dynamics models. Later, Hamer presented the mass-action principle [21,22] when studying the recurring epidemics of measles. A.G. McKendrick and W.O. Kermack formulated a famous modern mathematical epidemic model named the Susceptible-Infected-Recovered (SIR) compartmental model when studying the spreading pattern of the Black Death and the plague in 1906. SIR compartmental model successfully predicted the outbreak of several epidemics [23]. Harding et al. [24] followed the maximum entropy (MaxEnt) principle when simulating on the SIS model to study epidemics spreading on networks. Wang et al. [25] used entropy to incorporate many related factors, and designed a rumor spreading model. Zenil et al. [26] used algorithmic information content to steer the controlling of complex systems. By analyzing airline network data, Brockmann et al. identified the origin place of epidemics [27]. Pastor et al. summarized the methods and models in propagation dynamics [13], providing theoretical support for predicting and controlling propagation.

Identifying a set of influential spreaders in complex networks plays a crucial part in target advertisement, epidemics and rumor control. One naive method is to select top *k* ranked nodes as spreaders according to ranking methods. Lately, there have been many ranking methods to this problem. Some methods consider node’s local information, such as degree centrality, and its extending measures [28]. Degree centrality counts the number of node’s direct neighbors as its influence. H-index [29] considers 2-nd order neighbors as node’s importance. Based on degree centrality, LocalRank [30] takes node’s 4-th order neighbors into account. ClusterRank [31] combines Degree centrality and clustering coefficient [32] to measure node’s importance in spreading. Coreness centrality [33] decomposes graph by *K*-shell method, and it selects nodes based on their location within the graph. Zeng and Zhang [34] presented algorithm to rank nodes by decomposing networks in a mixed degree decomposition procedure. There are also some path-based methods to quantify nodes’ importance, such as Eccentricity centrality [35], Closeness centrality [36], Betweenness centrality [37], and Katz centrality [38]. These methods suffer from relatively high computational cost for calculating shortest path between nodes. Considering the mutual enhancement [39], researchers put forward Eigenvector centrality [40] and cumulative nomination algorithm [41]. Besides, random walk based methods have also been proposed, such as the famous PageRank [42] and LeaderRank [43]. Information entropy has also been used as importance indicator to measure nodes’ importance [44,45]. Qiao et al. proposed entropy centrality [46,47] to measure the potential for communication activity between node pair. Ai assumes that the removal of a more important node is likely to cause more structural variation. So he measured node importance by entropy variation [48] which calculates the change of graph entropy when that node is removed. In order to capture a wider range and greater abundance of information, Li et al. put forward entropy and mutual information-based centrality approach (EMI) [49] to take both topological and digital network characteristics into account. Fei et al. used relative entropy [50] to choose appropriate centrality measures when dealing with different networks. Zareie et al. [51] ranked nodes in social networks based on nodes’ spreading capability by information entropy. Shetty et al. [52] used entropy to detect influential nodes in low-level, incomplete and noisy social network. By combining the advantages of degree centrality, efficiency centrality, betweenness centrality and correlation centrality through entropy, Fan et al. proposed correlation centrality index [53] to quantify node’s importance. Recently, entropy has also been used to rank nodes in weighted networks [54] and social networks [55]. However, the node set built by simply assembling the nodes and sorting them employed by the aforementioned methods may not be comparable to an elaborately selected set of nodes due to the rich club phenomenon [56], namely, important nodes tend to overlap with each other. Thus, lots of methods aim to directly select a set of nodes are proposed.

Kempe et al. defined the problem of identifying a set of influential spreaders in complex networks as influence maximization problem [57], and they used hill-climbing based greedy algorithm that is within 63% of optimal in several models. Greedy method [58] is usually taken as the approximate solution of influence maximization problem, but it is not efficient for its high computational cost. Chen et al. [58] proposed NewGreedy and MixedGreedy method. Borgatti [59] specified mining influential spreaders in social networks by two classes: KPP-POS and KPP-NEG, based on which he calculated the importance of nodes. Narayanam et al. [60] proposed SPIN algorithm based on Shapley value to deal with information diffusion problem in social networks. Although the above greedy based methods can achieve relatively better result, they would cost lots of time for Monte Carlo simulation. So more heuristic algorithms were proposed. Chen et al. put forward simple and efficient DegreeDiscount algorithm [58] in which if one node is selected, its neighbors’ degree would be discounted. Zhang et al. proposed VoteRank [61] which selects the influential node set via a voting strategy. Zhao et al. [62] introduced coloring technology into complex networks to seperate independent node sets, and selected nodes from different node sets, ensuring selected nodes are not closely connected. Hu et al. [63] and Guo et al. [64] further considered the distance between independent sets and achieved a better performance. Bao et al. [65] sought to find dispersive distributed spreaders by a heuristic clustering algorithm. Zhou [66] proposed an algorithm to find a set of influential nodes via message passing theory. Ji el al. [67] considered percolation in the network to obtain a set of distributed and coordinated spreaders. Researchers also seek to maximize the influence by studying communities [68,69,70,71,72,73]. Zhang [74] seperated graph nodes into communities by using K-medoid method before selecting nodes. Gong et al. [75] divided graph into communities of different sizes, and selected nodes by using degree centrality and other indicators. Chen et al. [76] detected communities by using SHRINK and Kcut algorithm. Later they selected nodes from different communities as candidate nodes, and used CDH method to find final *k* influential nodes. Recently, some novel methods based on node dynamics have been proposed which rank nodes to select influential spreaders [77,78]. Şirag Erkol et al. made a systematic comparison between methods focused on influence maximization problem [79]. They classify multiple algorithms to three classes, and made a detailed explanation and comparison between methods. More algorithms in this domain are described and classified clearly by Lü et al. in their review paper [80].

Most of the non-greedy strategy methods suffer from a possibility that some spreaders are so close that their influence may overlap. DegreeDiscount and VoteRank use iterative selection strategy. After a node is selected, they weaken its neighbors’ influence to cope with the rich club phenomenon. However, these two algorithms roughly induce nodes’ local information. Besides, they do not further make use of the difference between nodes when weakening nodes’ influence. In this paper, we propose a new heuristic algorithm named EnRenew based on node’s entropy to select a set of influential nodes. EnRenew also uses iterative selection strategy. It initially calculates the influence of each node by its information entropy (further explained in Section 2.2), and then repeatedly select the node with the largest information entropy and renovate its *l*-length reachable nodes’ information entropy by an attenuation factor until specific number of nodes are selected. Experiments show that the proposed method yields the largest final affected scale on 6 real networks in the Susceptible-Infected-Recovered (SIR) simulation model compared with state-of-the-art benchmark methods. The results reveal that EnRenew could be a promising tool for related work. Besides, to make the algorithm practically more useful, we provide EnRenew’s source code and all the experiments details on https://github.com/YangLiangwei/Influential-nodes-identification-in-complex-networks-via-information-entropy, and researchers can download it freely for their convenience.

The rest of paper is organized as follows: The identifying method is presented in Section 2. Experiment results are analyzed and discussed in Section 3. Conclusions and future interest research topics are given in Section 4.

## 2. Methods

### 2.1. Spreading Model

The best way to measure the influence of a set of nodes in complex networks is through propagation dynamic process on real life network data. A susceptible infected removed model (SIR model) is initially used to simulate the dynamic of disease spreading [23]. It is later widely used to analyze similar spreading process, such as rumor [81] and population [82]. In this paper, the SIR model is adopted to objectively evaluate the spreading ability of nodes selected by algorithms. Each node in the SIR model can be classified into one of three states, namely, Susceptible nodes (S), Infected nodes (I), and Recovered nodes (R). At first, set initial selected nodes to infected status and all others in network to susceptible status. In each propagation iteration, each infected node randomly choose one of its direct neighbors and infect it with probability μ. In the meantime, each infected node will be recovered with probability β and won’t be infected again. In this study, $\lambda =\frac{\mu }{\beta }$ is defined as infected rate, which is crucial to the spreading speed in the SIR model. Apparently, the network can reach a steady stage with no infection after enough propagation iterations. To enable information spreads widely in networks, we set $\mu =1.5\mu_{c}$, where $\mu_{c}=\frac{\langle k\rangle }{\langle k^{2}\rangle -\langle k\rangle }$ [83] is the spreading threshold of SIR, 〈k〉 is the average degree of network. When μ is smaller than $\mu_{c}$, spreading in SIR could only affect a small range or even cannot spread at all. When it is much larger than $\mu_{c}$, nearly all methods could affect the whole network, which would be meaningless for comparison. Thus, we select μ around $\mu_{c}$ on the experiments. During the SIR propagation mentioned above, enough information can be obtained to evaluate the impact of initial selected nodes in the network and the metrics derived from the procedure is explained in Section 2.4.

### 2.2. EnRenew Algorithm

The influential nodes selecting algorithm proposed in this paper is named EnRenew, deduced from the concept of the algorithm. EnRenew introduces entropy and renews the nodes’ entropy through an iterative selection process. EnRenew is inspired by VoteRank algorithm proposed by Zhang et al. [61], where the influential nodes are selected in an iterative voting procedure. VoteRank assigns each node with voting ability and scores. Initially, each node’s voting ability to its neighbors is 1. After a node is selected, the direct neighbors’ voting ability will be decreased by $\frac{1}{\langle k\rangle }$, where $\langle k\rangle =\frac{2*m}{n}$ is the average degree of the network. VoteRank roughly assigns all nodes in graph with the same voting ability and attenuation factor, which ignores node’s local information. To overcome this shortcoming, we propose a heuristic algorithm named EnRenew and described as follows.

**Figure 1:**
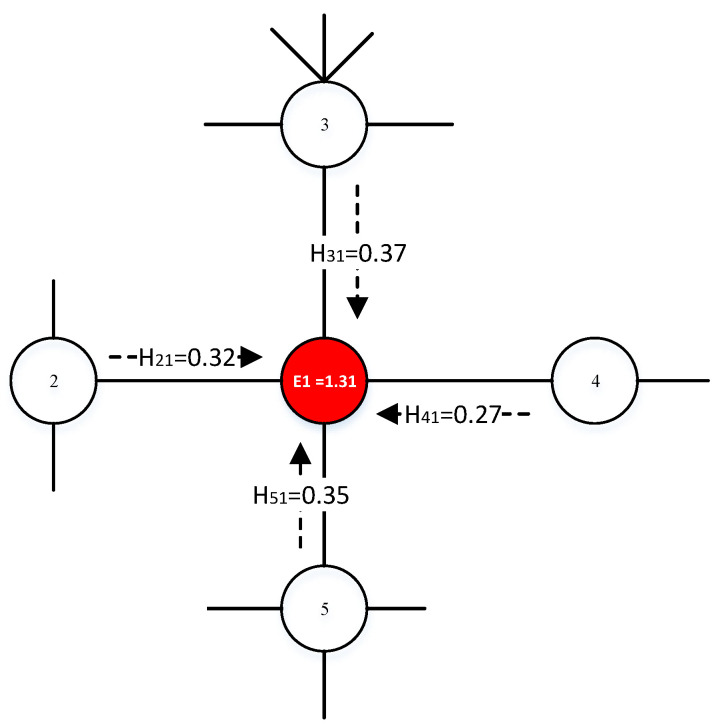
It shows how the red node’s (node 1) entropy is calculated in detail. Node 1 has four neighbors from node 2 to node 5. Node 1’s information entropy is then calculated by $E_{1}=H_{21}+H_{31}+H_{41}+H_{51}=0.32+0.37+0.27+0.35=1.31$.

In information theory, information quantity measures the information brought about by a specific event and information entropy is the expectation of the information quantity. These two concepts are introduced into complex network in Reference [44,45,46] to calculate the importance of node. Information entropy of any node *v* can be calculated by:

(1) $$E_{v}=\sum_{u\in \Gamma_{v}}H_{uv}=\sum_{u\in \Gamma_{v}}-p_{uv}\log p_{uv},$$

where $p_{uv}=\frac{d_{u}}{\sum_{l\in \Gamma_{v}}d_{l}}$, $\sum_{l\in \Gamma_{v}}p_{lv}=1$, $\Gamma_{v}$ indicates node *v*’s direct neighbors, and $d_{u}$ is the degree of node *u*. $H_{uv}$ is the spreading ability provided from *u* to *v*. $E_{v}$ is node *v*’s information entropy indicating its initial importance which would be renewed as described in Algorithm 1. A detailed calculating of node entropy is shown in Figure 1.
**Algorithm 1:** EnRenew
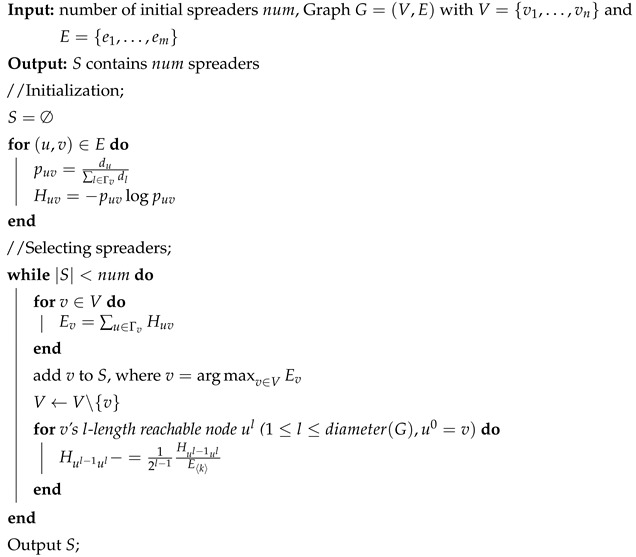


Simply selecting the nodes with a measure of degree as initial spreaders might not achieve good results. Because most real networks have obvious clumping phenomenon, that is, high-impact nodes in the network are often connected closely in a same community. Information cannot be copiously disseminated to the whole network. To manage this situation, after each high impact node is selected, we renovate the information entropy of all nodes in its local scope and then select the node with the highest information entropy, the process of which is shown in Algorithm 1.

$E_{\langle k\rangle }=-\langle k\rangle \cdot \frac{1}{\langle k\rangle }\cdot \log\frac{1}{\langle k\rangle }$ and 〈k〉 is the average degree of the network. $\frac{1}{2^{l-1}}$ is the attenuation factor, the farther the node is from node *v*, the smaller impact on the node will be. $E_{\langle k\rangle }$ can be seen as the information entropy of any node in 〈k〉-regular graph if 〈k〉 is an integer.

From Algorithm 1, we can see that after a new node is selected, the renew of its *l*-length reachable nodes’ information entropy is related with *H* and $E_{\langle k\rangle }$, which reflects local structure information and global network information, respectively. Compared with VoteRank, EnRenew replaces voting ability by *H* value between connected nodes. It induces more local information than directly set voting ability as 1 in VoteRank. At the same time, EnRenew uses $\frac{H}{E_{\langle k\rangle }}$ as the attenuate factor instead of $\frac{1}{\langle k\rangle }$ in VoteRank, retaining global information.

### 2.3. Computational Complexity Analysis

Computational complexity (usually time complexity) is used to describe the relationship between the input of different scales and the running time of the algorithm. Generally, brute force can solve most problems accurately, but it cannot be applied in most scenarios because of its intolerable time complexity. Time complexity is an extremely important indicator of an algorithm’s effectiveness. Through analysis, the algorithm is proved to be able to identify influential nodes in large-scale network in limited time. The computational complexity of EnRenew can be analyzed in three parts, initialization, selection and renewing. *n*, *m* and *r* represent the number of nodes, edges and initial infected nodes, respectively. At start, EnRenew takes O(n·〈k〉)=O(m) for calculating information entropy. Node selection selects the node with the largest information entropy and requires O(n), which can further be decreased to O(logn) if stored in an efficient data structure such as red-black tree. Renewing the *l*-length reachable nodes’ information entropy needs $O\left({\langle k\rangle }^{l}\right)=O\left(\frac{m^{l}}{n^{l}}\right)$. As suggested in Section 3.3, l=2 yields impressive results with $O(\frac{m^{2}}{n^{2}})$. Since selection and renewing parts need to be performed *r* times to get enough spreaders, the final computational complexity is $O\left(m+n\right)+O\left(r\log n\right)+O\left(r{\langle k\rangle }^{2}\right)=O\left(m+n+r\log n+\frac{rm^{2}}{n^{2}}\right)$. Especially, when the network is sparse and r≪n, the complexity will be decreased to O(n).

### 2.4. Performance Metrics

The algorithm’s performance is measured by the selected nodes’ properties including its spreading ability and location property. Spreading ability can be measured by infected scale at time *t* F(t) and final infected scale $F(t_{c})$, which are obtained from SIR simulation and widely used to measure the spreading ability of nodes [61,84,85,86,87,88]. $L_{S}$ is obtained from selected nodes’ location property by measuring their dispersion [61].

Infected scale F(t) demonstrates the influence scale at time *t* and is defined by

(2) $$F\left(t\right)=\frac{n_{I(t)}+n_{R(t)}}{n},$$

where $n_{I(t)}$ and $n_{R(t)}$ are the number of infected and recovered nodes at time *t*, respectively. At the same time step *t*, larger F(t) indicates more nodes are infected by initial influential nodes, while a shorter time *t* indicates the initial influential nodes spread faster in the network.

$F(t_{c})$ is the final affected scale when the spreading reaches stable state. This reflects the final spreading ability of initial spreaders. The larger the value is, the stronger the spreading capacity of initial nodes. $F(t_{c})$ is defined by:

(3) $$F\left(t_{c}\right)=\frac{n_{R(t_{c})}}{n},$$

where $t_{c}$ is the time when SIR propagation procedure reaches its stable state.

$L_{S}$ is the average shortest path length of initial infection set *S*. Usually, with larger $L_{S}$, the initial spreaders are more dispersed and can influence a larger range. This can be defined by:

(4) $$L_{S}=\frac{1}{\left|S|(|S|-1\right)}\sum_{u,v\in S,u\neq v}l_{u,v},$$

where $l_{u,v}$ denotes the length of the shortest path from node *u* to *v*. If *u* and *v* is disconnected, the shortest path is replaced by $D_{GC}$ + 1, where $D_{GC}$ is the largest diameter of connected components.

## 3. Results and Discussions

### 3.1. An Example Network

**Figure 2:**
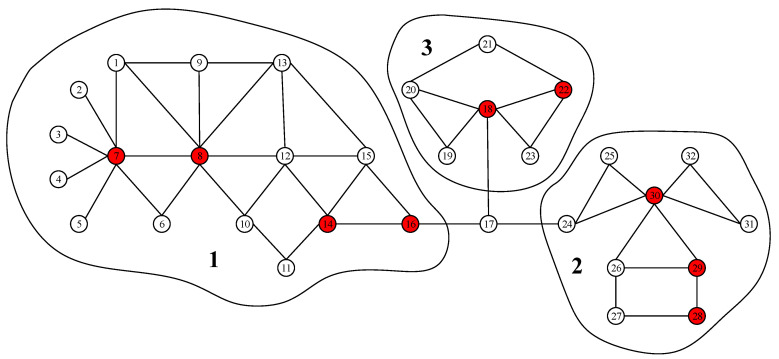
This network consists of three communities at different scales. The first nine nodes selected by EnRenew are marked red. The network typically shows the rich club phenomenon, that is, nodes with large degree tend to be connected together.

An example network shown in Figure 2 is used to show the rationality of nodes the proposed algorithm chooses. The first three nodes selected by EnRenew is distributed in three communities, while those selected by the other algorithms are not. We further run the SIR simulation on the example network with EnRenew and other five benchmark methods. The detailed result is shown in Table 1 for an in-depth discussion. This result is obtained by averaging 1000 experiments.

Table 2 shows the experiment results when choosing 9 nodes as the initial spreading set. Greedy method is usually used as the upper bound, but it is not efficient in large networks due to its high time complexity. EnRenew and PageRank distribute 4 nodes in community 1, 3 nodes in community 2, and 1 node in community 3. The distribution matches the size of community. However, the nodes selected by the other algorithms tend to cluster in community 1 except for greedy method. This will induce spreading within high density area, which is not efficient to spread in the entire network. EnRenew and PageRank can adaptively allocate reasonable number of nodes based on the size of the community just as Greedy method. Nodes selected by EnRenew have the second largest average distance except Greedy, which indicates EnRenew tends to distribute nodes sparsely in the graph. It aptly alleviates the adverse effect of spreading caused by the rich club phenomenon. Although EnRenew’s average distance is smaller than PageRank, it has a higher final infected scale $F(t_{c})$. Test result on PageRank also indicates that just select nodes widely spread across the network may not induce to a larger influence range. EnRenew performs the closest to Greedy with a low computational cost. It shows the proposed algorithm’s effectiveness to maximize influence with limited nodes.

### 3.2. Data Description

Table 2 describes six different networks varying from small to large-scale, which are used to evaluate the performance of the methods. CEnew [89] is a list of edges of the metabolic network of C.elegans. Email [90] is an Email user communication network. Hamster [91] is a network reflecting friendship and family links between users of the website http://www.hamsterster.com, where node and edge demonstrate the web user and relationship between two nodes, respectively. Router network [92] reflects the Internet topology at the router level. Condmat (Condense Matter Physics) [93] is a collaboration network of authors of scientific papers from the arXiv. It shows the author collaboration in papers submitted to Condense Matter Physics. A node in the network represents an author, and an edge between two nodes shows the two authors have collaboratively published papers. In the Amazon network [94], each node represents a product, and an edge between two nodes represents two products were frequently purchased together.

### 3.3. Analysis of Influence Range When Renewing

**Figure 3:**
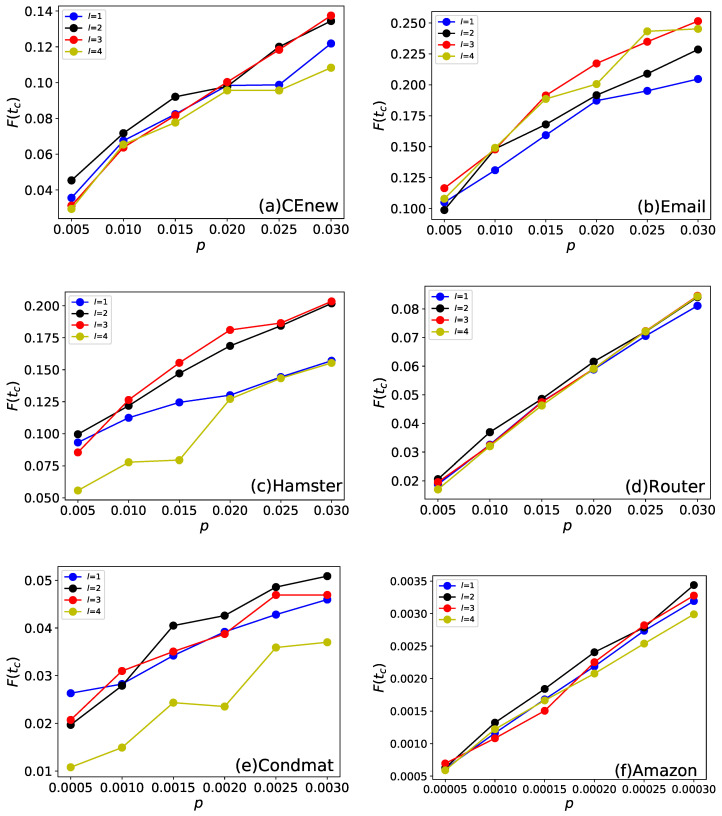
The figure shows how EnRenew’s parameter *l* influences final affected scale $F(t_{c})$ on the six networks. Each subfigure shows experiment results on one network. *p* is the ratio of initial infected nodes. The results are obtained by averaging on 100 independent runs with spread rate λ=1.5 in SIR. With specific ratio of initial infected nodes *p*, larger final affected scale $F(t_{c})$ means more reasonable of the parameter *l*. The best parameter *l* differs from different networks. In real life application, *l* can be used as an tuning parameter.

We firstly conduct experiments on the parameter *l*, which is the influence range when renewing the information entropy. If l=1, only the direct neighbors’ importance of selected node will be renewed, and if l=2, the importance of 2-length reachable nodes will be renewed and so forth. The results with varying parameter *l* from 1 to 4 on four networks are shown in Figure 3.

It can be seen from Figure 3 that, when l=2, the method gets the best performance in four of the six networks. In network Email, although the results when l=3 and l=4 are slightly better comparing with the case of l=2, the running time increases sharply. Besides, the three degrees of influence (TDI) theory [95] also states that a individual’s social influence is only within a relatively small range. Based on our experiments, we set the influence range parameter *l* at 2 in the preceding experiments.

### 3.4. Comparation with Benchmark Algorithms

Many factors affect the final propagation scale in networks. A good influential nodes mining algorithm should prove its robustness in networks varying in structure, nodes size, initial infection set size, infection probability, and recovery probability. To evaluate the performance of EnRenew, VoteRank, Adaptive Degree, *k*-shell, PageRank, and h-index algorithms are selected as benchmark methods for comparing. Furthermore, greedy method is usually taken as upper bound on influence maximization problem, but it is not practical on large networks due to its high time computational complexity. Thus, we added Greedy method as upper bound on the two small networks (CEnew and Email).

**Figure 4:**
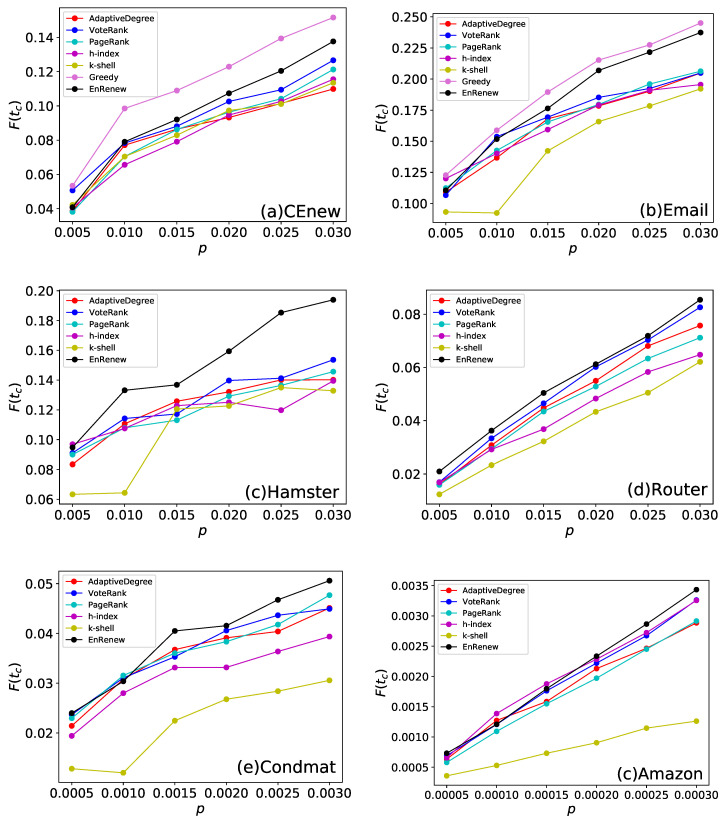
This experiment compares different methods by final affected scale $F(t_{c})$ on the six networks. Each subfigure shows experiment results on one network. *p* is the ratio of initial infected nodes. The results are obtained by averaging on 100 independent runs with spread rate λ=1.5 in Susceptible-Infected-Recovered (SIR). With specific ratio of initial spreading nodes *p*, larger final affected scale $F(t_{c})$ indicates that the selected nodes are more advantageous to spreading. It can be seen that EnRenew surpasses all the other benchmark methods on the six networks. On the two small network, EnRenew nearly reaches the upper bound.

The final affected scale $F(t_{c})$ of each method on different initial infected sizes are shown in Figure 4. It can be seen that EnRenew achieves an impressing result on the six networks. In the small network, such as CEnew and Email, EnRenew has an apparent better result on the other benchmark methods. Besides, it nearly reaches the upper bound on Email network. In Hamster network, it achieves a $F(t_{c})$ of 0.22 only by ratio of 0.03 initial infected nodes, which is a huge improvement than all the other methods. In Condmat network, the number of affected nodes are nearly 20 times more than the initial ones. In a large Amazon network, 11 nodes will be affected on average for one selected initial infected node. But the algorithm performs unsatisfactory on network Router. All the methods did not yield good results due to the high sparsity structure of the network. In this sparse network, the information can hardly spread out with small number of initial spreaders. By comparing the 6 methods from the Figure 4, EnRenew surpasses all the other methods on five networks with nearly all kinds of *p* varying from small to large. This result reveals that when the size of initial infected nodes varies, EnRenew also shows its superiority to all the other methods. What is worth noticing is that EnRenew performs about the same as other methods when *p* is small, but it has a greater improvement with the rise of initial infected ratio *p*. This phenomenon shows the rationality of the importance renewing process. The renewing process of EnRenew would influence more nodes when *p* is larger. The better improvement of EnRenew than other methods shows the renewing process reasonability redistributes nodes’ importance.

**Figure 5:**
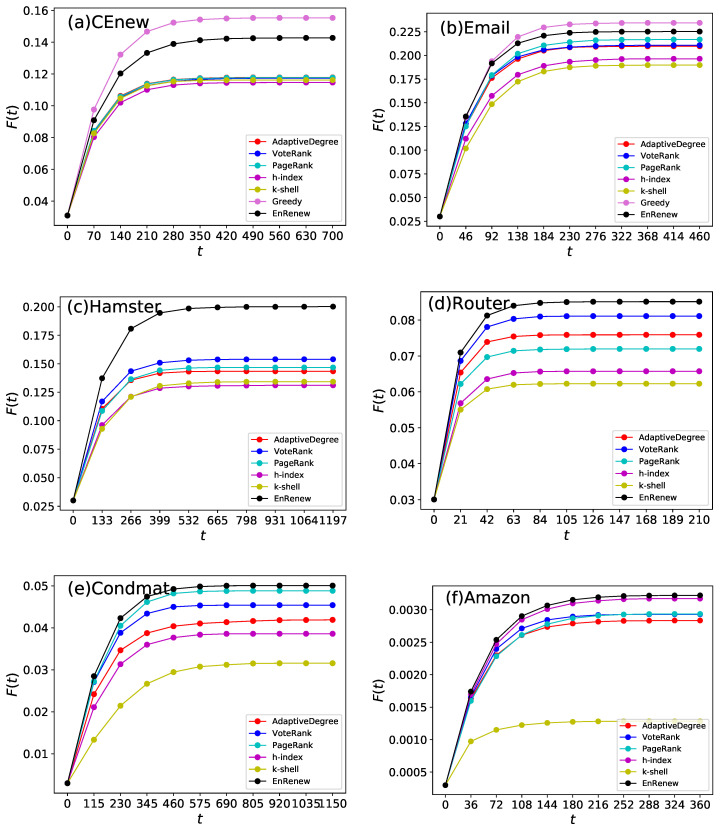
This experiment compares different methods regard to spreading speed. Each subfigure shows experiment results on one network. The ratio of initial infected nodes is 3% for CEnew, Email, Hamster and Router, 0.3% for Condmat and 0.03% for Amazon. The results are obtained by averaging on 100 independent runs with spread rate λ=1.5 in SIR. With the same spreading time *t*, larger F(t) indicates larger influence scale in network, which reveals a faster spreading speed. It can be seen from the figures that EnRenew spreads apparently faster than other benchmark methods on all networks. On the small network CEnew and Email, EnRenew’s spreading speed is close to the upper bound.

Timestep experiment is made to assess the propagation speed when given a fixed number of initial infected nodes. The exact results of F(t) varying with time step *t* are shown in Figure 5. From the experiment, it can be seen that with same number of initial infected nodes, EnRenew always reaches a higher peak than the benchmark methods, which indicates a larger final infection rate. In the steady stage, EnRenew surpasses the best benchmark method by 21.1%, 7.0%, 30.0%, 5.0%, 2.5% and 9.0% in final affected scale on CEnew, Email, Hamster, Router, Condmat and Amazon networks, respectively. In view of propagation speed, EnRenew reaches the peak at about 300th time step in CEnew, 200th time step in Email, 400th time step in Hamster, 50th time step in Router, 400th time step in Condmat and 150th time step in Amazon. EnRenew always takes less time to influence the same number of nodes compared with other benchmark methods. From Figure 5, it can also be seen that *k*-shell also performs worst from the early stage in all the networks. Nodes with high core value tend to cluster together, which makes information hard to dissipate. Especially in the Amazon network, after 100 timesteps, all other methods reach a F(t) of 0.0028, which is more than twice as large as *k*-shell. In contrast to *k*-shell, EnRenew spreads the fastest from early stage to the steady stage. It shows that the proposed method not only achieve a larger final infection scale, but also have a faster infection rate of propagation.

**Figure 6:**
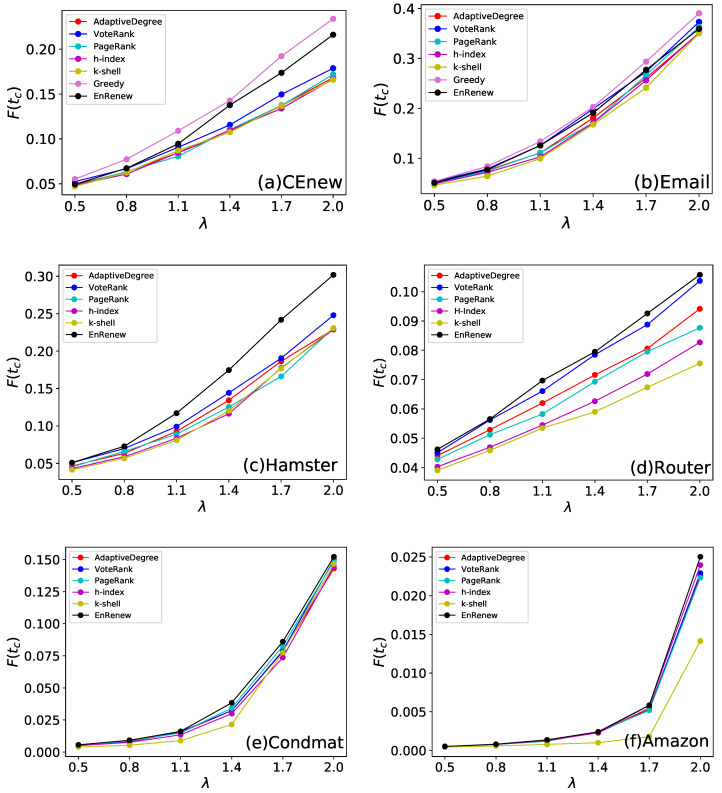
This experiment tests algorithms’ effectiveness on different spreading conditions. Each subfigure shows experiment results on one network. The ratio of initial infected nodes is 3% for CEnew, Email, Hamster and Router, 0.3% for Condmat, and 0.03% for Amazon. The results are obtained by averaging on 100 independent runs. Different infected rate λ of SIR can imitate different spreading conditions. EnRenew gets a larger final affected scale $F(t_{c})$ on different λ than all the other benchmark methods, which indicates the proposed algorithm has more generalization ability to different spreading conditions.

In real life situations, the infected rate λ varies greatly and has huge influence on the propagation procedure. Different λ represents virus or information with different spreading ability. The results on different λ and methods are shown in Figure 6. From the experiments, it can be observed that in most of cases, EnRenew surpasses all other algorithms with λ varying from 0.5 to 2.0 on all networks. Besides, experiment results on CEnew and Email show that EnRenew nearly reaches the upper bound. It shows EnRenew has a stronger generalization ability comparing with other methods. Especially, the EnRenew shows its impressing superiority in strong spreading experiments when λ is large.

**Figure 7:**
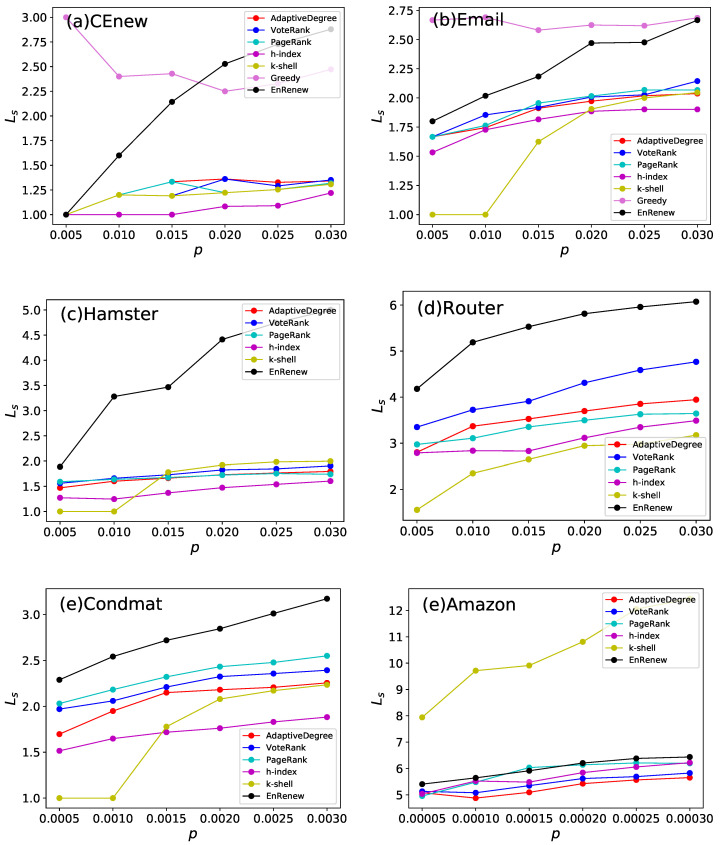
This experiment analysis average shortest path length $L_{S}$ of nodes selected by different algorithms. Each subfigure shows experiment results on one network. *p* is the ratio of initial infected nodes. Generally speaking, larger $L_{S}$ indicates the selected nodes are more sparsely distributed in network. It can be seen that nodes selected by EnRenew have the apparent largest $L_{S}$ on five networks. It shows EnRenew tends to select nodes sparsely distributed.

Generally speaking, if the selected nodes are widely spread in the network, they tend to have an extensive impact influence on information spreading in entire network. $L_{S}$ is used to measure dispersity of initial infected nodes for algorithms. Figure 7 shows the results of $L_{S}$ of nodes selected by different algorithms on 6 different networks. It can be seen that, except for the Amazon network, EnRenew always has the largest $L_{S}$, indicting the widespread of selected nodes. Especially in CEnew, EnRenew performs far beyond all the other methods as its $L_{S}$ is nearly as large as the upper bound. In regard to the large-scale Amazon network, the network contains lots of small cliques and *k*-shell selects the dispersed cliques, which makes *k*-shell has the largest $L_{S}$. But other experimental results of *k*-shell show a poor performance. This further confirms that EnRenew does not naively distribute selected nodes widely across the network, but rather based on the potential propagation ability of each node.

## 4. Conclusions

The influential nodes identification problem has been widely studied by scientists from computer science through to all disciplines [96,97,98,99,100]. Various algorithms that have been proposed aim to solve peculiar problems in this field. In this study, we proposed a new method named EnRenew by introducing entropy into a complex network, and the SIR model was adopted to evaluate the algorithms. Experimental results on 6 real networks, varying from small to large in size, show that EnRenew is superior over state-of-the-art benchmark methods in most of cases. Besides, with its low computational complexity, the presented algorithm can be applied to large scale networks. The EnRenew proposed in this paper can also be well applied in rumor controlling, advertise targeting, and many other related areas. But, for influential nodes identification, there still remain many challenges from different perspectives. From the perspective of network size, how to mine influential spreaders in large-scale networks efficiently is a challenging problem. In the area of time-varying networks, most of these networks are constantly changing, which poses the challenge of identifying influential spreaders since they could shift with the changing topology. In the way of multilayer networks, it contains information from different dimensions with interaction between layers and has attracted lots of research interest [101,102,103]. To identify influential nodes in multilayer networks, we need to further consider the method to better combine information from different layers and relations between them.

## Figures and Tables

**Table 1:** Experiment results on example network shown in Figure 2. The first three nodes selected by EnRenew are distributed in three communities. The best results of Community Distribution, Average Distance and $F(t_{c})$ are marked in bold.

Method	Initial Spreading Nodes									Community Distribution			Average Distance	$F(t_{c})$
	1	2	3	4	5	6	7	8	9	1	2	3		
Adaptive Degree [58]	8	30	7	18	12	16	13	26	24	5	3	1	3.64	18.22
PageRank [42]	7	8	30	18	12	13	26	29	22	**4**	**3**	**2**	4.33	17.56
h-index [29]	18	12	13	17	9	8	30	16	15	6	1	1	2.75	15.64
*k*-shell [33]	7	8	30	18	12	13	10	26	17	5	2	1	3.72	17.97
VoteRank [61]	7	30	8	18	12	14	26	22	12	5	2	2	3.94	18.36
Greedy [58]	17	6	27	21	16	32	1	11	23	4	2	2	**4.92**	**21.33**
EnRenew (l=2)	**8**	**30**	**18**	7	16	22	14	28	29	**4**	**3**	**2**	4.27	18.81

**Table 2:** Topological features of networks.

Networks	*n*	*m*	〈k〉	$k_{\max}$	〈c〉
CEnew	453	2025	8.94	237	0.646
Email	1133	5451	9.62	71	0.22
Hamster	2426	16631	13.711	273	0.538
Router	5022	6258	2.492	106	0.012
Condmat	23133	93497	8.083	281	0.633
Amazon	334863	925872	5.530	549	0.397

Note: *n* and *m* are the total number of nodes and edges, respectively, and $\langle k\rangle =\frac{2*m}{n}$ stands for average node degree and $k_{max}=\max_{v\in V}d_{v}$ is the max degree in the network and Average clustering coefficient 〈c〉 measures the degree of aggregation in the network. $\langle c\rangle =\frac{1}{n}\sum_{i=1}^{n}\frac{2*I_{i}}{|\Gamma_{i}\left|*(\right|\Gamma_{i}\left|-1\right)}$, where $I_{i}$ denotes the number of edges between direct neighbors of node *i*.
